# A Comparative Pharmacokinetic Study by UHPLC-MS/MS of Main Active Compounds after Oral Administration of Zushima-Gancao Extract in Normal and Adjuvant-Induced Arthritis Rats

**DOI:** 10.3390/molecules23010227

**Published:** 2018-01-22

**Authors:** Jinjun Shan, Wenjuan Qian, Linxiu Peng, Lianghui Chen, An Kang, Tong Xie, Liuqing Di

**Affiliations:** 1 Jiangsu Key Laboratory of Pediatric Respiratory Disease, Institute of Pediatrics, Nanjing University of Chinese Medicine, Nanjing 210023, China; jshan@njucm.edu.cn (J.S.); kanga@njucm.edu. (A.K.); sunnyxyl1021@163.com (T.X.); 2 State Key Laboratory Cultivation Base for TCM Quality and Efficacy, School of Pharmacy, Nanjing University of Chinese Medicine, Nanjing 210023, China; 20161325@njucm.edu.cn (W.Q.); penglinxiu1016@hotmail.com (L.P.); chenlhnow@126.com (L.C.); 3 Jiangsu Key Laboratory for Functional Substance of Chinese Medicine, Nanjing 210023, China

**Keywords:** Zushima-Gancao extract, UHPLC-MS/MS, rheumatoid arthritis, pharmacokinetics

## Abstract

A sensitive and rapid ultra high-performance liquid-chromatography tandem mass spectrometry (UHPLC-MS/MS) method has been applied to investigate the influence of rheumatoid arthritis (RA) on the pharmacokinetics of nine analytes (daphnetin, daphnoretin, 7-hydroxycoumarin, liquiritin, isoliquiritin, liquiritigenin, isoliquiritigenin, glycyrrhizin, and glycyrrhetinic acid), which are major active components in Zushima-Gancao extract. The analytes and internal standard (IS) were separated in a Hypersil Gold C_18_ column and detected on a triple-stage quadrupole mass spectrometer using the validated method. All analytes exhibited good linearities (*R*^2^ > 0.98), and the lower limit of quantification (LLOQs) were sufficient for quantitative analysis. Intra- and inter-batch precision were all within 14.96% while the accuracy of nine analytes ranged from −17.99 to 14.48%, and these results were all within acceptance criteria. The extraction recoveries, matrix effects, and stabilities were all satisfactory. Main pharmacokinetic parameters of each compound were compared, and significant differences were found in parameters of daphnetin, daphnoretin, liquiritin, isoliquiritin, isoliquiritigenin, glycyrrhizin, and glycyrrhetinic acid, especially the last one, between the two groups. Therefore, adjuvant-induced arthritis has different effects on the pharmacokinetics of ingredients in Zushima-Gancao extract. The comparative pharmacokinetic study between normal and adjuvant-induced arthritis rats might provide more comprehensive information to guide the clinical usage of Zushima-Gancao extract for treating RA.

## 1. Introduction

Rheumatoid arthritis (RA) is a chronic systemic disease accompanied by the destruction of joints, but its etiology has remained unknown [1]. Western medicine, including nonsteroidal anti-inflammatory drugs (NSAIDS) [2] and biologics [3], has greatly improved the clinical efficacy of medicine acting against RA. However, the adverse reactions and toxicities associated with these drugs [4,5,6] have promoted the development of traditional Chinese herbal medicine (TCHM), such as *Semen strychni*, *Tripterygium wilfordii*, and *Daphne giraldii* Nitsche. These TCHMs are featured with high pharmacological activities and have been widely used to treat RA for decades [7,8]. 

Zushima is the root cortex and shoot bark of *Daphne giraldii* Nitsche, *D. tangutica* Maxim, and *D. retusa* Hemsl. In the past several decades, much more attention has been paid to the chemical and biological studies of Zushima [7,9]. It was reported that coumarins and diterpenes were the main bioactive components and the former has function of anti-inflammation and painkilling [10,11,12]. Gancao was derived from the dried roots and rhizomes of the Glycyrrhiza species, and it was clinically used to treat wounds, diabetes, cough, and tuberculosis [13]. Additionally, it also exhibited an anti-inflammatory effect [14]. Furthermore, Gancao was reported to have a distinct synergistic effect when combined with other Chinese medicines, such as Jiegeng [15] and Fuzi [16].

Previous studies revealed that the efficacy of Zushima was significantly increased; meanwhile, the stimulus was reduced in concomitant use with Gancao [17,18]. Our group has found that the bioavailability of Zushima could be improved obviously when the ratio of Zushima and Gancao reaches 3:2 [19]. Furthermore, our research also investigated the mechanism of Zushima-Gancao extract in treating rheumatoid arthritis based on plasma untargeted metabolomics and found that Zushima-Gancao extract was able to regulate potential biomarkers in rat plasma, such as valine, phenylalanine, and palmitic acid [20]. A great number of studies have proved that daphnetin (DPH), daphnoretin (DR) and 7-hydroxycoumarin (HDC) are categorized as coumarins with relative high concentration in Zushima, while liquiritin (LQ), liquiritigenin (LG), isoliquiritin (ILQ), isoliquiritigenin (ILG), and glycyrrhizin (GL) are the primary ingredients extracted from Gancao [21,22]. These compounds have been validated to have anti-arthritis and anti-inflammatory effects. For instance, DPH was reported to augment Th17 cells and inhibit Treg cells to reduce the swelling and inflammation in the feet of CIA (Collagen induced Arthritis) rats [23], while DR could inhibit osteoclast activity and promote osteoblast proliferation, two important cells that maintain the balance of bone metabolism [24].

It was reported that lower drug metabolism was associated with decreased hepatic cytochromes P450 (CYPs) owing to pathological states, especially those involving a host inflammation [25]. In terms of RA, hepatic CYP1A, CYP3A, and CYP2B subfamily enzymes was found to decrease during the development of arthritis and was accompanied by a increase in tumor necrosis factor (TNF-α) and interleukin-1β (IL-1β) [26,27]. The metabolism of liquiritigenin and glycyrrhetinic acid were mediated by CYP1A and CYP3A, respectively, and glycyrrhetinic acid (GA) could be formed from hydrolysis of glycyrrhizin in intestinal flora according to this summary [28,29], while the effect of CYPs on the compounds in Zushima was not reported. In summary, it is essential to emphasize the pathological state in clinical medication. The adjuvant-induced arthritis (AA) model was applied to our experiment because it could replicate the characteristics of RA in human. Moreover, the mechanisms of RA could be investigated using this model [30,31].

In this study, we developed a simple, rapid, and reliable UHPLC-MS/MS method for the simultaneous determination of daphnetin, daphnoretin, 7-hydroxycoumarin, liquiritin, liquiritigenin, isoliquiritin, isoliquiritigenin, glycyrrhizin, and glycyrrhetinic acid in rat plasma. This is the first time the comparative pharmacokinetic profiles of analytes mentioned above, after oral administration of Zushima-Gancao extract in normal and adjuvant-induced arthritis rats, have been investigated.

## 2. Results and Discussion

### 2.1. Method Validation

#### 2.1.1. Specificity

**Figure 1:**
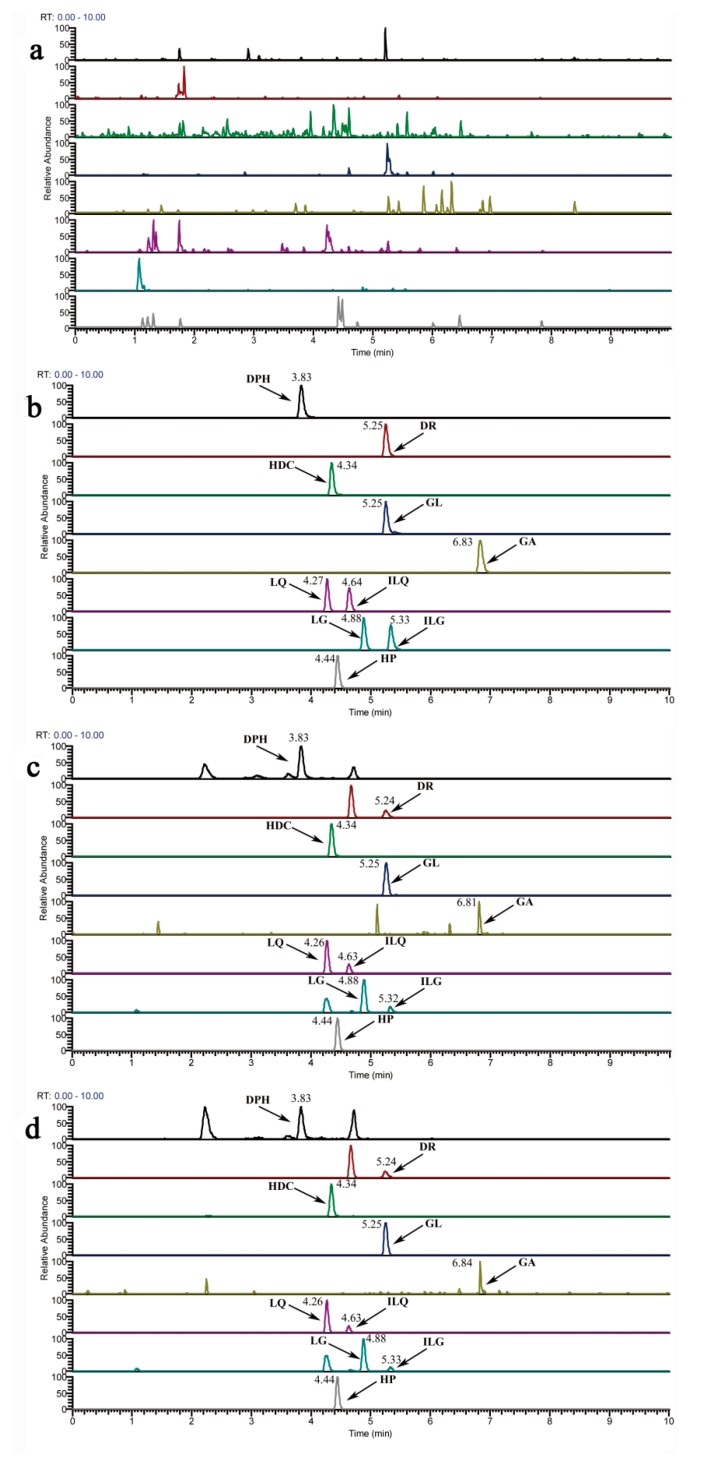
The selective reaction monitor (SRM) chromatograms of analytes in blank plasma samples (**a**); blank plasma samples spiked with mixed standards and IS (**b**); plasma samples obtained from normal rats (**c**); and plasma samples obtained from AA model rats (**d**). Abbreviation notes: DPH: daphnetin; DR: daphnoretin; HDC: 7-hydroxycoumarin; LQ: liquiritin; LG: liquiritigenin; ILQ: isoliquiritin; ILG: isoliquiritigenin; GL: glycyrrhizin; GA: glycyrrhetinic acid.

No significant endogenous interference was observed at the retention times of the analytes and IS from the chromatograms of blank plasma samples (Figure 1a), blank plasma samples spiked with mixed standards and IS (Figure 1b), plasma samples obtained from normal rats (Figure 1c), and plasma samples obtained from AA rats (Figure 1d) collected from different rats (six in each group) after administration of Zushima-Gancao extract.

#### 2.1.2. Linearity and LLOQ

The results of the calibration curve, linear range, correlation coefficient (*R*^2^), and LLOQ of each compound are shown in Table 1, and all analytes exhibited good linearity within the test ranges.

#### 2.1.3. Accuracy and Precision

The accuracy and precision results are summarized in Table 2. The intra-batch and inter-batch precision of the nine analytes were all in the range of 2.12–14.96% and 1.92–13.63%. The accuracy values ranged from −14.96 to 14.48% for intra-batch and from −17.99 to 14.44% for inter-batch. The results demonstrated that the accuracy and precision of the nine analytes were well within the acceptable limit.

#### 2.1.4. Recovery and Matrix Effect

The results showed that the recoveries of each ingredient at low, medium, and high concentrations were over 60%, even if the recovery of IS was slightly lower. It was proved that the liquid-liquid extraction with ethyl acetate could be applied for extracting measured components.

Matrix effect values of nine analytes ranged from 85.54 to 114.66%, and the matrix effect of IS was 96.23%. The results indicated that the endogenous matrix peaks did not affect the quantification of all the analytes and IS. Recovery and matrix effect data of nine analytes and IS in rat plasma are shown in Table 3.

#### 2.1.5. Stability

The results of stability are shown in Table 4, and all ingredients were stable at 4 °C for 24 h, at room temperature for 4 h, at −80 °C for a week, and three freeze-thaw cycles.

### 2.2. Pharmacokinetics

**Figure 2:**
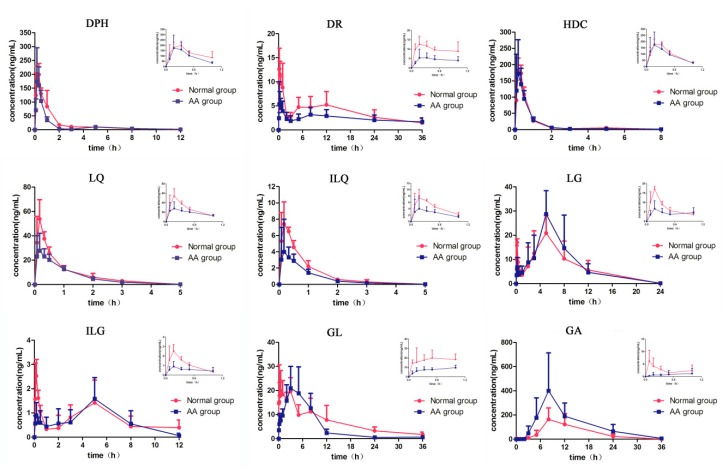
Time–concentration profiles of nine components in plasma from normal (●) and AA (■) rats after oral administration of Zushima-Gancao extract. The upper right corner of each drug curve shows the corresponding drug profile within 1 h. Abbreviation notes: DPH: daphnetin; DR: daphnoretin; HDC: 7-hydroxycoumarin; LQ: liquiritin; ILQ: isoliquiritin; LG: liquiritigenin; ILG: isoliquiritigenin; GL: glycyrrhizin; GA: glycyrrhetinic acid.

The validated method was applied to the pharmacokinetic study of DPH, DR, HDC, LQ, ILQ, LG, ILG, GL, and GA in normal and AA rats after oral administration of Zushima-Gancao extract. The plasma concentration–time curves (C-T curves) of nine analytes are presented in Figure 2, and the corresponding pharmacokinetic parameters, including *C*_max_, *T*_max_, AUC (0*–t*), AUC (0–∞) (AUC: area under concentration-time curve), and *t*_1/2_, were obtained using DAS 3.0 pharmacokinetic software (Chinese Pharmacological Association, China). Statistical differences between groups were analyzed using Student’s *t*-tests, and *p*-values less than 0.05 were considered statistically significant. All data are summarized in Table 5.

According to the pharmacokinetic results, it was found that the plasma profile of LG and ILG presented bimodal (double peak) phenomenon after oral administration of Zushima-Gancao extract in both normal and AA rats. This phenomenon was because LG, as well as ILG, were conjugated with glucuronic acid in the liver, and the conjugations were hydrolyzed in the intestinal tract and then re-absorbed into the blood [32]. Interestingly, there was a similar tendency in the pharmacokinetic profiles of three pairs of structurally similar compounds (i.e., DPH and HDC, LQ and ILQ, and LG and ILG), which possibly exhibited the same absorbing and metabolic features.

Furthermore, it was noticed that the pharmacokinetic parameters of components in AA rats were significantly different from those in normal rats, especially the AUC (0–∞) of DPH in Zushima and four compounds (LQ, ILQ, GL, and GA) in Gancao. Compared with the normal group, the AUC (0–∞) of DPH, LQ, ILQ, and GL obviously decreased by 34.56%, 24.57%, 39.02%, and 29.89%, respectively. The parameters also showed that the *C*_max_ of DR, LQ and ILQ decreased remarkably (*p* < 0.05). On the contrary, the bioavailability of LG and GA increased; the AUC(0*–t*) and AUC (0–∞) of GA especially were both increased 2.2-fold in the model rats compared to those in normal rats. In our previous investigation, the in vitro concentration of DPH in Zushima-Gancao extract was 6-fold compared to DR and 22-fold compared to HDC, but DPH had the worst in vivo utilization according to the AUC of the three coumarins. That was probably because DPH was partially metabolized into HDC. In terms of GA, some previous published papers have reported that GL was hydrolyzed to GA by *Ruminococcus* sp.POI-3, *Eubacterium* sp., and β-d-glucuronidase in the intestinal tract [33]. The possible explanation for the difference between two groups could be that the characteristics of intestinal flora have changed in AA rats, and more GL was metabolized into GA.

The *t*_1/2_ value of DPH exhibited a remarkable decrease in AA rats; meanwhile, an obvious increase of *T*_max_ values for ILQ, ILG, and GL were observed in AA rats compared with normal rats. This phenomenon revealed that the disease had different effects on the pharmacokinetic behaviors of different compounds.

## 3. Materials and Methods 

### 3.1. Materials and Reagents

Zushima (batch number 20120812) was obtained from Shanxi Pharmaceutical Co., Ltd. (Xi’an, China). Gancao (batch number 130101) was obtained from Anhui Wan sheng Chinese Medicine Yinpian Co., Ltd. (Fuyang, China). The plant materials are identified as *Daphne giraldii* Nitsche and *Glcyrrhiza glabra*, respectively, by Dr. Shengjin Liu (Department of Pharmacy, Nanjing University of Chinese Medicine, Nanjing, China).

**Figure 3:**
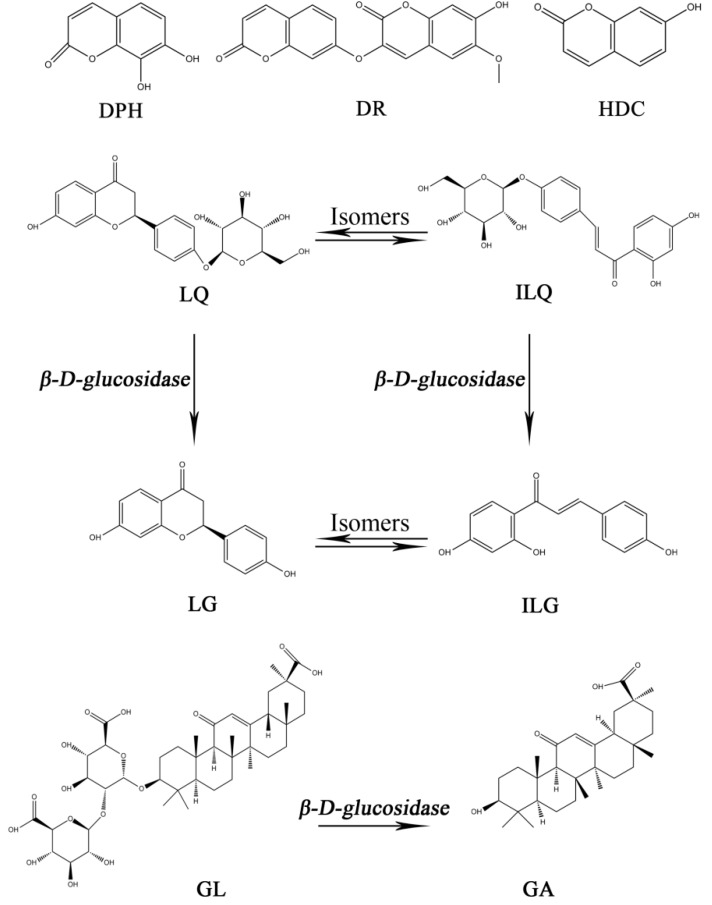
The chemical structures of nine components in Zushima and Gancao and the relationship between them are annotated in the figure as appropriate. Abbreviation notes: DPH: daphnetin; DR: daphnoretin; HDC: 7-hydroxycoumarin; LQ: liquiritin; ILQ: isoliquiritin; LG: liquiritigenin; ILG: isoliquiritigenin; GL: glycyrrhizin; GA: glycyrrhetinic acid.

Reference standard materials of daphnetin (DPH) (batch number 121025), daphnoretin (DR) (batch number 130305), 7-hydroxycoumarin (HDC) (batch number 120927), liquiritin (LQ) (batch number 111127), isoliquiritin (ILQ) (batch number 201010), liquiritigenin (LG) (batch number 201004), isoliquiritigenin (ILG) (batch number 101026), glycyrrhizin (GL) (batch number 111023), and glycyrrhetinic acid (GA) (batch number 110906) were all purchased from Sichuan Weikeqi Bio-tech Co., Ltd. (Chengdu, China). The chemical structures of these compounds are shown in Figure 3. Hesperidin (HP) (batch number 0721-200010) used as an internal standard (IS) was purchased from the National Institute for the Control of Pharmaceutical and Biological Products (Beijing, China). All standard compounds were determined by HPLC and their purities were all higher than 98%.

Bacille calmette-guerin (BCG) (batch number 20110928) was purchased from National Vaccine and Serum Institute (Beijing, China). Liquid paraffin (batch number 20070901) was purchased from Shanghai Jiuyi Reagent Co., Ltd. (Shanghai, China). Lanolin (batch number 20081201) was purchased from Shanghai Jingxi Chemical Technology Co., Ltd. (Shanghai, China). Heparin sodium injections (batch number 121010) were purchased from Changzhou Qianhong Bio-pharma Co., Ltd. (Changzhou, China). Ethyl acetate (batch number 13030110243) was purchased from Nanjing Chemical Reagent Co., Ltd. (Nanjing, China).

Acetonitrile and methanol (HPLC grade) were obtained from Merck (Darmstadt, Germany). Formic acid with a purity of 99% was of HPLC grade obtained from ROE Scientific (St. Louis, MO, USA). Deionized water was purified using a Milli-Q purification system (Millipore, Milford, MA, USA). Other reagents and chemicals were of analytical grade.

### 3.2. Instrument and Analytical Conditions

The chromatography experiment was carried out using a UHPLC system (Thermo Fisher, San Jose, CA, USA), equipped with a Hypersil GOLD C18 column (100 mm × 2.1 mm, 3 μm, Thermo Fisher, San Jose, CA, USA) maintained at 35 °C. The mobile phase consisted of 0.05% formic acid in water (Solvent A) and acetonitrile (Solvent B), and the flow rate was 0.3 mL/min. In order to completely separate all the analytes, the 10 min gradient elution program was used as follows: 0–1 min, 10% B; 1–5 min, 10–90% B; 5–6 min, 90–100% B; 6–8 min, 100% B; 8–9 min, 100–10% B; 9–10 min, 10% B, and the sample injection volume was 3 μL.

Mass spectrometry detection was operated on TSQ Vantage triple-stage quadrupole mass spectrometer (Thermo Fisher, San Jose, CA, USA) equipped with an electrospray ionization (ESI) source. Quantification of the analytes was performed using selective reaction monitor (SRM) in the negative ionization mode. Spray voltage, sheath gas pressure, auxiliary gas pressure, vaporizer temperature, and capillary temperature were set as 3.0 kV, 45 arb, 25 arb, 450 °C, and 350 °C, respectively. The optimized SRM parameters of nine analytes and IS are listed in Table 6.

### 3.3. Preparation of Zushima-Gancao Extract

For preparing Zushima-Gancao extract, the herbal materials composed of Zushima (162 g) and Gancao (108 g) were crushed into small pieces and mixed, and the mixture was then soaked in water (1:10, *w/v*) for 0.5 h before decocting for 2 h. The filtrates were collected and the residues were then refluxed in water (1:5, *w/v*) for 1 h. Zushima-Gancao extract (equal to 0.162 g/mL of Zushima and 0.108 g/mL of Gancao) could be obtained by mixing the two-stage filtrates and concentrating the volume to 1000 mL. The concentrations of DPH, DR, HDC, LQ, ILQ, LG, ILG, and GL in prescription were 220.60, 36.56, 10.25, 507.30, 56.48, 42.03, 3.39, and 1006.00 μg/mL respectively. GA was not detected in the extract.

### 3.4. Preparation of Standard Solutions and Quality Control (QC) Samples

Stock solutions were separately dissolved in methanol to concentrations of 1.27 mg/mL for DPH, 1.56 mg/mL for DR, 1.03 mg/mL for HDC, 1.20 mg/mL for LQ, 1.02 mg/mL for ILQ, 1.10 mg/mL for LG, 1.05 mg/mL for ILG, 1.08 mg/mL for GL, and 1.12 mg/mL for GA, and they were then serially diluted with methanol to prepare working solutions with 10 standards of desired concentrations. Hesperidin(IS) was dissolved in 0.5 mL of DMSO and diluted with methanol to achieve a concentration of 130 ng/mL. All the solutions were stored at 4 °C.

The calibration standards of DPH (1.65–843.33 ng/mL), DR (0.09–46.20 ng/mL), HDC (0.80–410.00 ng/mL), LQ (0.59–300.00 ng/mL), ILQ (0.50–255.00 ng/mL), LG (0.36–183.33 ng/mL), ILG (0.34–175.00 ng/mL), GL (1.06–540.00 ng/mL), and GA (4.92–2520.00 ng/mL) were prepared by spiking 100 μL of blank plasma with a 10 μL working solution.

Quality control (QC) samples were obtained in the same way as calibration standards with blank plasma in low, middle, and high concentrations (3.29, 52.71, 421.67 ng/mL for DPH; 0.18, 2.89, 23.10 ng/mL for DR; 1.60, 25.63, 205.00 ng/mL for HDC; 1.17, 18.75, 150.00 ng/mL for LQ; 1.00, 15.94, 127.50 ng/mL for ILQ; 0.72, 11.46, 91.67 ng/mL for LG; 0.68, 10.94, 87.50 ng/mL for ILG; 2.11, 33.75, 270.00 ng/mL for GL; 9.84, 157.50, 1260.00 ng/mL for GA).

### 3.5. Animals and Induction of FAC

Male Sprague–Dawley (SD) rats weighing 180–220 g (SCXK-2012-0002) were supplied by Silaike Lab Animals Ltd. (Shanghai, China). They were all kept in a stable condition (temperature, 22 ± 2 °C; relative humidity, 45–60%) with 12 h light/dark cycles for five days before animal experiments.

Rats were randomly divided into normal and AA groups with six rats in each group. The arthritic model rats were prepared as our previous research [19]. AA rats were intracutaneously injected with 0.1 mL of FAC in the left hind footpad and normal rats were injected with sterile saline. The FAC solution was prepared as follows: bacillus calmette-guerin (BCG) was bathed in water at 80 °C for 30 min, and then dissolved in a liquid paraffin–lanolin mixture (3:2, *v/v*). Immediately prior to use, the FAC solution were sterilized for 30 min at 120 °C.

### 3.6. Drug Administration and Blood Sampling

After model construction, the rats were fed for 20 days. All rats were fasted for 12 h before drug administration, with free access to water. Each rat was administered with Zushima-Gancao extract at a single dose of 10 mL/kg. Blood samples (approximately 0.3 mL) were collected into heparinized micro-centrifuge tubes from the postorbital venous plexus veins of rats at specific time points of 0, 0.08, 0.17, 0.33, 0.5, 1, 2, 3, 5, 8, 12, 24, and 36 h. All blood samples were centrifuged immediately at 5000 rpm for 6 min, and the plasma were obtained and stored at −80 °C until analysis.

### 3.7. Plasma Sample Preparation

A liquid–liquid extraction (LLE) method was applied to extract the compounds from thawed rat plasma samples, aliquots of 100 μL plasma sample were transferred into a 1.5 mL Eppendorf tube, and 10 μL of methanol, 10 μL of hydrochloric acid (2 mol/L), and 10 μL of IS (130 ng/mL) were added separately. The mixture was vortexed for 30 s, and 500 μL of ethyl acetate was added and mixed by vortexing for 10 min. The supernatant (about 450 μL) was transferred into another tube after centrifugation at 16,000 rpm for 10 min and then evaporated to dryness at 45 °C, 15 kPa in a Thermo SPD1010-230 SpeedVac Concentrator (Thermo Fisher, San Jose, CA, USA). The residue was dissolved in 100 μL of initial mobile phase by vortexing for 5 min, and a further centrifugation at 17,000 rpm for 10 min was applied after the residue was re-dissolved. The supernatant was injected into the UHPLC-MS/MS system for analysis.

### 3.8. Method Validation

The method was validated in accordance with The Guidance for Industry—Bioanalytical Method Validation for specificity, lower limit of quantification (LLOQ), linearity, accuracy, precision, recovery, matrix effect, and stability.

**Specificity.** The specificity was assessed to ensure no endogenous interference. It was carried out by comparing the chromatograms of blank plasma samples, blank plasma samples spiked with mixed standards and IS, plasma samples obtained from normal rats, and plasma samples obtained from AA rats collected from different rats (six in each group) after administration of Zushima-Gancao extract.

**Linearity and LLOQ.** The samples for calibration curves were prepared by spiking the blank rat plasma (100 μL) with 10 μL of the working solutions at different concentrations before extracting the analytes. The linearities of the calibration curves were determined by plotting the peak-area ratios of analytes/IS (Y) against concentration (X) with a weighted (*W* = 1/*X*) linear least squares regression model. The lower limit of quantification (LLOQ) was defined as the lowest concentration point of the standard curve with a signal-to-noise (S/N) ratio >10 and a precision and accuracy within ±20%.

**Precision and accuracy**. The QC samples and the sample at the concentration of LLOQ were analyzed with six replicates at each concentration to determine intra-batch and inter-batch accuracy (expressed as relative error, RE%) and precision (expressed as relative standard deviation, RSD%) in the same day or over three consecutive days.

**Extraction recovery and matrix effect**. Extraction recovery tests of nine analytes and IS were performed by comparing the peak areas of QC samples and IS added prior to extraction of the plasma samples with the peak areas of pure standards at the same concentration spiked with extracted plasma. The matrix effect was evaluated by comparing the peak areas of post-extracted blank samples spiked with analytes at three concentration levels, as well as IS with the same concentration of analytes, and IS dissolved in initial mobile phase.

**Stability**. To evaluate the stability of each component during analysis, QC samples at low, middle, and high concentrations (three samples for each concentration) were tested under different conditions, including placing QC samples in the autosampler at 4 °C for 24 h, at room temperature for 4 h, at −80 °C for a week, and three freeze-thaw cycles.

## 4. Conclusions

Based on a sensitive and rapid UHPLC-MS/MS method, nine analytes, three coumarins in Zushima and six compounds in Gancao, were simultaneously determined in normal and AA rat plasma samples after oral administration of Zushima-Gancao extract. The pharmacokinetic parameters between the two groups were compared and the results suggested that the pathological state could influence the pharmacokinetic characteristics of compounds in prescription. The bioavailabilities of DPH, LQ, ILQ, and GL were significantly decreased and GA increased when the rats were in a pathological state. In general, AA is a suitable model for simulating organisms with rheumatoid arthritis, and the different pharmacokinetics of nine ingredients between normal and AA rats revealed that the bioavailability of drugs is influenced by the internal environment when organisms are in a pathological state.

## Figures and Tables

**Table 1:** Calibration curves, linear ranges, correlation coefficients (*R*^2^) and LLOQ of DPH, DR, HDC, LQ, ILQ, LG, ILG, GL, and GA in rat plasma.

Analytes	RT (min)	Calibration Curves	*R*^2^	Liner Range (ng/mL)	LLOQ (ng/mL)
DPH	3.83	*Y* = 0.0058*x* − 0.0051	0.992	1.65–843.33	1.65
DR	5.24	*Y* = 0.0985*x* + 0.0036	0.998	0.09–46.20	0.09
HDC	4.34	*Y* = 0.0509*x* + 0.0189	0.995	0.80–410.00	0.80
LQ	4.26	*Y* = 0.0349*x* + 0.0064	0.998	0.59–300.00	0.59
ILQ	4.63	*Y* = 0.0408*x* − 0.0003	0.999	0.50–255.00	0.50
LG	4.88	*Y* = 0.0420*x* + 0.0072	0.998	0.36–183.33	0.36
ILG	5.33	*Y* = 0.0664*x* + 0.0010	0.998	0.34–175.00	0.34
GL	5.25	*Y* = 0.0076*x −* 0.0032	0.997	1.06–540.00	1.06
GA	6.84	*Y* = 0.0030*x* + 0.0016	0.986	4.92–2520.00	4.92

Abbreviation notes: DPH: daphnetin; DR: daphnoretin; HDC: 7-hydroxycoumarin; LQ: liquiritin; LG: liquiritigenin; ILQ: isoliquiritin; ILG: isoliquiritigenin; GL: glycyrrhizin; GA: glycyrrhetinic acid.

**Table 2:** Intra-batch and inter-batch accuracy and precision of LC-MS/MS determination of DPH, DR, HDC, LQ, ILQ, LG, ILG, GL, and GA in rat plasma.

Analytes	Spiked Conc. (Concentration) (ng/mL)	Intra-Batch			Inter-Batch		
		Measured Conc. (ng/mL)	Accuracy (RE, %)	Precision (RSD, %)	Measured Conc. (ng/mL)	Accuracy (RE, %)	Precision (RSD, %)
DPH	1.65	1.53 ± 0.22	−6.92	14.24	1.76 ± 0.14	7.33	7.66
	3.29	3.19 ± 0.46	−3.17	14.42	3.24 ± 0.17	−1.8	5.32
	52.71	60.34 ± 4.54	14.48	7.52	59.84 ± 3.31	13.52	5.53
	421.67	470.39 ± 51.80	11.56	11.01	467.53 ± 31.31	10.88	6.70
DR	0.09	0.08 ± 0.01	−12.68	13.76	0.09 ± 0.01	−3.51	7.18
	0.18	0.17 ± 0.02	−6.5	12.22	0.16 ± 0.01	−11.95	7.59
	2.89	3.17 ± 0.33	9.81	10.51	2.82 ± 0.33	−2.32	11.69
	23.10	24.22 ± 3.05	4.86	12.57	20.18 ± 0.61	−12.63	3.03
HDC	0.80	0.73 ± 0.11	−9.22	14.93	0.80 ± 0.09	0	10.90
	1.60	1.50 ± 0.12	−6.58	7.86	1.60 ± 0.08	−0.28	4.89
	25.63	28.06 ± 0.94	9.49	3.34	27.33 ± 1.05	6.67	3.83
	205.00	225.27 ± 17.64	9.89	7.83	206.31 ± 5.42	0.64	2.63
LQ	0.59	0.52 ± 0.06	−10.76	11.20	0.52 ± 0.07	−11.52	13.49
	1.17	1.18 ± 0.08	0.23	7.11	1.23 ± 0.03	5.08	2.42
	18.75	20.80 ± 0.70	10.92	3.37	20.38 ± 0.39	8.67	1.92
	150	152.62 ± 4.09	1.75	2.68	148.79 ± 4.37	−0.8	2.93
ILQ	0.50	0.42 ± 0.02	−14.96	4.59	0.41 ± 0.02	−17.99	4.31
	1.00	1.04 ± 0.11	4.31	10.46	1.10 ± 0.10	10.86	8.66
	15.94	16.71 ± 0.52	4.83	3.11	16.49 ± 0.42	3.47	2.57
	127.50	114.79 ± 2.43	−9.97	2.12	114.25 ± 2.49	−10.4	2.18
LG	0.36	0.31 ± 0.04	−12.56	13.15	0.31 ± 0.05	−12.88	14.50
	0.72	0.72 ± 0.09	−0.03	12.41	0.67 ± 0.06	−7	9.59
	11.46	12.45 ± 0.83	8.69	6.65	11.57 ± 0.32	1.01	2.75
	91.67	96.48 ± 6.61	5.25	6.85	89.12 ± 4.69	−2.77	5.26
ILG	0.34	0.33 ± 0.05	−3.79	14.47	0.36 ± 0.02	6.48	5.18
	0.68	0.75 ± 0.07	9.18	9.00	0.78 ± 0.05	14.44	6.02
	10.94	10.60 ± 1.55	−3.05	14.60	12.24 ± 0.26	11.92	2.16
	87.50	75.49 ± 9.20	−13.73	12.19	87.44 ± 4.65	−0.06	5.31
GL	1.06	1.02 ± 0.12	−2.98	11.77	0.92 ± 0.13	−13.03	13.63
	2.11	2.19 ± 0.33	8.49	14.96	2.13 ± 0.17	5.45	7.96
	33.75	30.74 ± 1.75	−8.91	5.69	28.94 ± 1.34	−14.25	4.62
	270.00	230.24 ± 20.08	−14.73	8.72	241.57 ± 29.73	−10.53	12.31
GA	4.92	4.26 ± 0.42	−13.55	9.76	4.37 ± 0.55	−11.17	12.60
	9.84	8.76 ± 0.94	−11.02	10.75	8.49 ± 0.87	−13.77	10.24
	157.50	153.85 ± 18.33	−2.31	11.92	177.70 ± 6.92	12.83	3.90
	1260.00	1390.83 ± 80.9	10.38	5.81	1413.66 ± 76.5	12.20	5.41

Abbreviation notes: DPH: daphnetin; DR: daphnoretin; HDC: 7-hydroxycoumarin; LQ: liquiritin; LG: liquiritigenin; ILQ: isoliquiritin; ILG: isoliquiritigenin; GL: glycyrrhizin; GA: glycyrrhetinic acid.

**Table 3:** The extraction recovery and matrix effect of DPH, DR, HDC, LQ, ILQ, LG, ILG, GL, GA, and HP in rat plasma.

Analytes	Spiked Conc. (ng/mL)	Extraction Recovery		Matrix Effect	
		Accuracy(%)	RSD (%)	Accuracy (%)	RSD (%)
DPH	3.29	69.56	12.53	109.47	11.90
	52.71	72.51	5.00	113.45	10.59
	421.67	79.09	7.37	114.66	2.36
DR	0.18	89.54	4.81	112.16	1.69
	2.89	82.73	10.52	105.43	3.01
	23.10	76.71	3.60	99.00	1.52
HDC	1.60	86.79	3.62	107.81	8.83
	25.63	84.35	3.14	106.78	1.43
	205.00	84.53	2.67	101.20	2.64
LQ	1.17	64.58	2.49	99.95	6.24
	18.75	61.50	2.53	102.39	1.89
	150.00	61.91	2.16	92.07	3.26
ILQ	1.00	74.46	10.65	96.94	7.39
	15.94	68.70	2.25	100.51	3.06
	127.50	60.50	1.68	97.23	0.80
LG	0.72	79.71	6.76	113.61	13.08
	11.46	82.75	2.55	106.29	2.28
	91.67	84.13	6.64	99.74	0.97
ILG	0.68	60.88	4.65	86.52	4.67
	10.94	60.11	1.89	88.90	3.69
	87.50	64.45	3.45	85.54	1.48
GL	2.11	78.43	9.73	113.73	8.27
	33.75	71.89	3.78	105.21	5.49
	270.00	72.50	11.11	97.11	7.39
GA	9.84	63.06	6.59	112.46	9.39
	157.50	62.43	2.51	85.65	4.21
	1260.00	62.59	6.21	86.18	5.93
HP	130.00	56.53	1.81	96.23	1.67

Abbreviation notes: DPH: daphnetin; DR: daphnoretin; HDC: 7-hydroxycoumarin; LQ: liquiritin; LG: liquiritigenin; ILQ: isoliquiritin; ILG: isoliquiritigenin; GL: glycyrrhizin; GA: glycyrrhetinic acid; HP: hesperidin(IS).

**Table 4:** The stability test of DPH, DR, HDC, LQ, ILQ, LG, ILG, GL, and GA in rat plasma.

Analytes	Spiked Conc. (ng/mL)	24 h Stability		Short-Term Stability		Long-Term Stability		Freeze-Thaw Stability	
		RE (%)	RSD (%)	RE (%)	RSD (%)	RE (%)	RSD (%)	RE (%)	RSD (%)
DPH	3.29	−13.85	5.82	−7.83	2.46	−12.07	12.53	−6.29	3.04
	52.71	−13.73	2.46	−12.68	2.48	−15	1.05	−8.25	9.42
	421.67	−11.8	1.01	−12.42	4.59	−12.23	4.78	−14.04	8.97
DR	0.18	−11.79	2.82	−13.63	13.16	−12.79	7.07	0.05	7.16
	2.89	−11.33	1.64	−13.99	1.55	−14.41	2.08	−14.22	2.82
	23.10	−6.11	2.00	−5.38	1.15	−13.85	0.91	−9.71	4.68
HDC	1.60	−1.43	8.39	−13.86	11.16	−10.07	1.85	−13.48	10.06
	25.63	−0.85	0.41	−5.72	1.54	−1.8	0.62	−1.27	3.13
	205.00	1.39	1.03	3.09	4.68	−0.05	2.08	2.64	2.00
LQ	1.17	2.03	7.34	−9.95	12.36	8.14	7.75	13.76	6.71
	18.75	3.31	6.83	0.14	4.83	−0.13	4.22	−5.95	3.17
	150.00	4.89	0.74	5.23	0.98	7.57	1.46	7.92	3.61
ILQ	1.00	4.99	8.75	9.08	2.98	7.49	1.72	−2.51	10.17
	15.94	5.33	5.05	12.35	3.29	5.37	5.51	−6.3	12.35
	127.50	6.64	1.21	3.29	1.67	2.66	0.63	4.51	4.30
LG	0.72	6.72	2.25	9.76	4.54	2.87	1.91	7.22	1.47
	11.46	8.15	10.56	11.16	8.65	0.58	3.69	7.96	7.41
	91.67	8.56	5.59	1.5	3.74	3.89	4.49	6.75	8.16
ILG	0.68	8.75	4.32	−3.23	9.06	−11.29	7.39	2.53	13.94
	10.94	10.08	0.72	11.76	0.61	12.68	2.32	12.3	6.33
	87.50	10.13	1.01	12.09	5.32	13.23	4.15	7.59	2.24
GL	2.11	11.79	3.24	10.26	0.34	11.48	2.49	10.89	4.87
	33.75	11.8	2.74	10.68	2.60	9.96	4.18	14.3	3.73
	270.00	13.22	3.31	13.85	1.02	9.2	2.20	14.36	2.99
GA	9.84	13.49	10.22	2.08	1.00	0.04	6.85	−14.06	5.84
	157.50	14.1	11.46	−3.38	5.79	5.44	3.31	4.21	11.57
	1260.00	14.35	0.98	13.05	2.43	9.57	2.15	13.8	2.27

Abbreviation notes: DPH: daphnetin; DR: daphnoretin; HDC: 7-hydroxycoumarin; LQ: liquiritin; LG: liquiritigenin; ILQ: isoliquiritin; ILG: isoliquiritigenin; GL: glycyrrhizin; GA: glycyrrhetinic acid.

**Table 5:** The pharmacokinetic parameters of DPH, DR, HDC, LQ, ILQ, LG, ILG, GL, and GA in normal and AA rats after oral administration of Zushima-Gancao extract to rats (*n* = 6, mean ± SD).

Analytes	Group	*C*_max_ (ng/mL)	*T*_max_ (h)	AUC_(0*–t*)_ (ng/mL·h)	AUC_(0–∞)_ (ng/mL·h)	*t*_1/2_ (h)
DPH	Normal	214.45 ± 32.55	0.24 ± 0.11	245.51 ± 53.86	255.68 ± 54.77	3.40 ± 0.96
	AA	200.83 ± 108.24	0.20 ± 0.07	164.51 ± 49.38	167.32 ± 49.04 *	2.06 ± 0.26 *
DR	Normal	14.40 ± 3.31	0.39 ± 0.31	131.35 ± 58.12	163.26 ± 78.67	14.53 ± 2.91
	AA	7.06 ± 3.82 *	0.42 ± 0.31	86.17 ± 33.84	153.80 ± 79.83	28.32 ± 9.39
HDC	Normal	193.15 ± 29.30	0.22 ± 0.08	141.91 ± 24.42	147.92 ± 24.89	2.86 ± 1.94
	AA	179.33 ± 98.00	0.22 ± 0.08	127.35 ± 26.80	130.73 ± 29.06	2.52 ± 1.82
LQ	Normal	58.75 ± 12.54	0.17 ± 0.09	45.45 ± 3.89	45.55 ± 3.90	0.47 ± 0.15
	AA	30.11 ± 13.83 **	0.21 ± 0.10	33.12 ± 6.80 **	34.36 ± 8.89 *	0.75 ± 0.65
ILQ	Normal	8.92 ± 1.68	0.17 ± 0.09	6.72 ± 0.60	6.74 ± 0.60	0.52 ± 0.20
	AA	4.63 ± 3.64 *	0.36 ± 0.13 *	4.04 ± 1.20 *	4.11 ± 1.12 **	0.94 ± 0.83
LG	Normal	23.95 ± 3.72	3.86 ± 1.98	169.46 ± 62.38	169.58 ± 62.49	1.66 ± 0.44
	AA	29.69 ± 10.69	4.70 ± 2.52	192.60 ± 83.23	193.82 ± 84.43	2.30 ± 0.95
ILG	Normal	2.77 ± 0.83	0.14 ± 0.05	8.89 ± 3.12	12.38 ± 6.97	1.08 ± 0.67
	AA	1.92 ± 0.55	3.70 ± 2.10 **	8.25 ± 2.90	8.48 ± 3.04	1.39 ± 0.65
GL	Normal	29.02 ± 10.49	0.58 ± 0.35	248.04 ± 47.75	266.68 ± 49.71	8.72 ± 4.81
	AA	24.95 ± 7.81	4.83 ± 2.64 *	183.66 ± 61.69	186.96 ± 64.62 *	4.88 ± 3.04
GA	Normal	167.00 ± 92.99	8.67 ± 1.63	1938.01 ± 1244.25	1959.74 ± 1261.64	3.88 ± 1.29
	AA	417.84 ± 296.20	9.33 ± 2.07	4258.35 ± 1862.33 *	4331.78 ± 1862.81 *	4.94 ± 2.43

* *p* < 0.05, ** *p* < 0.01 versus normal group. Abbreviation notes: DPH: daphnetin; DR: daphnoretin; HDC: 7-hydroxycoumarin; LQ: liquiritin; ILQ: isoliquiritin; LG: liquiritigenin; ILG: isoliquiritigenin; GL: glycyrrhizin; GA: glycyrrhetinic acid.

**Table 6:** The analytical conditions for the LC-MS/MS analysis of DPH, DR, HDC, LQ, ILQ, LG, ILG, GL, GA, and HP.

Analytes	M.W.	*m/z*	Collision Energy (V)	S-Lens
DPH	178.14	176.9→121.0	25	75
DR	352.29	351.0→162.9	39	102
HDC	162.14	160.9→133.0	21	77
GL	822.93	821.1→350.8	39	160
GA	470.68	469.1→425.2	37	160
LQ(ILQ)	418.39	417.1→255.0	22	92
LG(ILG)	256.25	255.0→135.0	18	65
HP	610.56	609.2→301.1	27	160

Abbreviation notes: DPH: daphnetin; DR: daphnoretin; HDC: 7-hydroxycoumarin; LQ: liquiritin; ILQ: isoliquiritin; LG: liquiritigenin; ILG: isoliquiritigenin; GL: glycyrrhizin; GA: glycyrrhetinic acid; HP: hesperidin(IS).

## References

[1] Qi Y., Li S., Pi Z., Song F., Lin N., Liu S., Liu Z. (2014). Metabonomic study of wu-tou decoction in adjuvant-induced arthritis rat using ultra-performance liquid chromatography coupled with quadrupole time-of-flight mass spectrometry. J. Chromatogr. B Anal. Technol. Biomed. Life Sci..

[2] Van Vollenhoven R.F. (2009). Treatment of rheumatoid arthritis: State of the art 2009. Nat. Rev. Rheumatol..

[3] Umekita K., Umeki K., Miyauchi S., Ueno S., Kubo K., Kusumoto N., Takajo I., Nagatomo Y., Okayama A. (2015). Use of anti-tumor necrosis factor biologics in the treatment of rheumatoid arthritis does not change human t-lymphotropic virus type 1 markers: A case series. Mod. Rheumatol..

[4] Cavagna L., Caporali R., Trifiro G., Arcoraci V., Rossi S., Montecucco C. (2013). Overuse of prescription and otc non-steroidal anti-inflammatory drugs in patients with rheumatoid arthritis and osteoarthritis. Int. J. Immunopathol. Pharmacol..

[5] Moller B., Pruijm M., Adler S., Scherer A., Villiger P.M., Finckh A. (2015). Chronic nsaid use and long-term decline of renal function in a prospective rheumatoid arthritis cohort study. Ann. Rheum. Dis..

[6] Tacheci I., Bradna P., Douda T., Bastecka D., Kopacova M., Rejchrt S., Lutonsky M., Soukup T., Bures J. (2016). Small intestinal injury in NSAID users suffering from rheumatoid arthritis or osteoarthritis. Rheumatol. Int..

[7] Li S.H., Wu L.J., Yin H.Y. (2002). Chemical and pharmacological advances of the study on zushima. China J. Chin. Mater. Med..

[8] Li Y., Wang J., Xiao Y., Wang Y., Chen S., Yang Y., Lu A., Zhang S. (2015). A systems pharmacology approach to investigate the mechanisms of action of semen strychni and tripterygium wilfordii hook f for treatment of rheumatoid arthritis. J. Ethnopharmacol..

[9] Yang G.H., Wang L.L., Feng L., Shi S.L. (2015). Evaluation on pharmacodynamics of analgesia and anti-inflammation of zushima coumarins. Chin. Arch. Tradit. Chin. Med..

[10] Su J., Wu Z., Shen Y., Liu R., Zhang C., Li H., Zhang W. (2008). Flavonoids from daphne giraldii nitsche. Nat. Prod. Res..

[11] Su J., Wu Z.J., Zhang W.D., Zhang C., Li H.L., Liu R.H., Shen Y.H. (2008). Two new bis-coumarin glycosides from *daphne giraldii* NITSCHE. Chem. Pharm. Bull..

[12] Huyiligeqi, Dong X., Yang C., Xu G., Cao S., Fu J., Lin L., Ni J. (2016). Chemical constituents from *daphne giraldii* Nitsche and their contents simultaneous determination by HPLC. Evid.-Based Complement. Altern. Med..

[13] Alrushaid S., Davies N.M., Martinez S.E., Sayre C.L. (2016). Pharmacological characterization of liquiritigenin, a chiral flavonoid in licorice. Res. Pharm. Sci..

[14] Ohno H., Araho D., Uesawa Y., Kagaya H., Ishihara M., Sakagami H., Yamamoto M. (2013). Evaluation of cytotoxiciy and tumor-specificity of licorice flavonoids based on chemical structure. Anticancer Res..

[15] Mao Y.C., Peng L.X., Kang A., Xie T., Xu J.Y., Shen C.S., Ji J.J., Di L.Q., Wu H., Shan J.J. (2017). Influence of jiegeng on pharmacokinetic properties of flavonoids and saponins in gancao. Molecules.

[16] Yang Y., Yin X.J., Guo H.M., Wang R.L., Song R., Tian Y., Zhang Z.J. (2014). Identification and comparative analysis of the major chemical constituents in the extracts of single fuzi herb and fuzi-gancao herb-pair by UFLC-IT-TOF/MS. Chin. J. Nat. Med..

[17] Meng X.L., Guo X.H., Zhang S.S. (2012). Research on processing mechanism of zushima which was stir-fried with licorice based on tg-dtg. China J. Chin. Mater. Med..

[18] Zhang W., Di L.Q., Li J.S., Shan J.J., Kang A., Qian S., Chen L.T. (2014). The effects of glycyrrhizae uralenis and its major bioactive components on pharmacokinetics of daphnetin in cortex daphnes in rats. J. Ethnopharmacol..

[19] Zhang W., Gong L., Zhou L.L., Shan J.J., Chen L.T., Xu H.Q., Di L.Q. (2014). Effect of different compatibility of daphnes giraldii cortex and glycyrrhizae radix et rhizoma on adjuvant-induced arthritis in rats. Chin. Tradit. Herb. Drugs.

[20] Peng L.X., Chen L.H., Di L.Q., Shan J.J., Xie T., Kang A., Xu N.S. (2017). Plasma metabonomic study on Zushima Gancao Tablet in treatment of rheumatoid arthritis based on UPLC/LTQ-Orbitrap-MS. Chin. Tradit. Herb. Drugs.

[21] Wang P., Liu J.P., Zhan N., Li Y.P., Lu D. (2011). Progress in the research on chemical constituents and pharmacological activities of zushima. Spec. Wild Econ. Anim. Plant Res..

[22] Shu K., Kuang N., Zhang Z., Hu Z., Zhang Y., Fu Y., Min W. (2014). Therapeutic effect of daphnetin on the autoimmune arthritis through demethylation of proapoptotic genes in synovial cells. J. Transl. Med..

[23] Huang J.F., Feng T.T., Lu Y., Zhang X., Chen L.Z., Zhou Y. (2016). Research on active of daphnoretin on inhibiting osteoclast differentiation and promoting osteoblast proliferation in vitro. Pharmacol. Clin. Chin. Mater. Med..

[24] Chen L.H., Shan J.J., Xie T., Di L.Q. (2014). Influence of zushima combined with gancao on dissolution of their eight components by LC-MS /MS. Chin. Tradit. Pat. Med..

[25] Morgan E.T. (1997). Regulation of cytochromes p450 during inflammation and infection. Drug Metab. Rev..

[26] Cornelis M.C., Bae S.C., Kim I., El-Sohemy A. (2010). Cyp1a2 genotype and rheumatoid arthritis in koreans. Rheumatol. Int..

[27] Sanada H., Sekimoto M., Kamoshita A., Degawa M. (2011). Changes in expression of hepatic cytochrome p450 subfamily enzymes during development of adjuvant-induced arthritis in rats. J. Toxicol. Sci..

[28] Ploeger B., Mensinga T., Sips A., Seinen W., Meulenbelt J., DeJongh J. (2001). The pharmacokinetics of glycyrrhizic acid evaluated by physiologically based pharmacokinetic modeling. Drug Metab. Rev..

[29] Liu H., Chen L.T., Yun F., Di L.Q., Shan J.J., Zhao X.L., Cai B.C. (2012). Research and thinking on characteristics and mechanisms of absorption and metabolism of glycyrrhiza radix et rhizoma and its compatible interactions with other herbs. Chin. Tradit. Herb. Drugs.

[30] Chen L.T., Jing Y.Y., Di L.Q., Xu H.Q., Wu H., Shan J.J. (2011). Study on the daphne giraldii nitsche. Effective parts of anti-rheumatoid arthritis. Res. Pract. Chin. Med..

[31] Xu T., Liu S., Zhao J., Feng G., Pi Z., Song F., Liu Z. (2015). A study on the effective substance of the wu-tou formula based on the metabonomic method using UPLC-Q-TOF-HDMS. Mol. BioSyst..

[32] Cheng J., Di L.Q., Shan J.J., Zhao X.L., Kang A., Bi X.L., Li J.S. (2014). Studies on effects of achyranthes bidentata on tongsaimai pellets main active ingredients chlorogenic acid, isoliquiritin, harpagoside and glycyrrhizin in vivo pharmacokinetics. China J. Chin. Mater. Med..

[33] Akao T. (2000). Effects of glycyrrhizin and glycyrrhetic acid on the growth, glycyrrhizin beta-d-glucuronidase and 3 beta-hydroxysteroid dehydrogenase of human intestinal bacteria. Biol. Pharm. Bull..

